# Benefits of a 12 week physical activity programme on muscle and bone health in people living with HIV

**DOI:** 10.1002/jcsm.12824

**Published:** 2021-10-01

**Authors:** Matteo Bonato, Laura Galli, Simona Bossolasco, Cecilia Bertocchi, Giuseppe Balconi, Marco Borderi, Pierluigi Viale, Gaspare Pavei, Giampiero Merati, Antonio La Torre, Adriano Lazzarin, Giuseppe Banfi, Paola Cinque

**Affiliations:** ^1^ Department of Biomedical Sciences for Health Università degli Studi di Milano Milan Italy; ^2^ IRCCS Istituto Ortopedico Galeazzi Milan Italy; ^3^ IRCCS San Raffaele Scientific Institute Milan Italy; ^4^ Infection Diseases Unit, Sant'Orsola Hospital University of Bologna Bologna Italy; ^5^ Department of Pathophysiology and Transplantation Università degli Studi di Milano Milan Italy; ^6^ Department of Biotechnology and Life Sciences (DBSV) University of Insubria Varese Italy; ^7^ Vita‐Salute San Raffaele University Milan Italy

Thanks to the use of combination antiretroviral treatment (cART) people living with HIV (PLWH) have a similar life expectancy to HIV‐negative people.^1^, ^2^ However, PLWH may be at higher risk to develop chronic diseases associated with persistent immune activation despite virological control.^3^ Physical activity has been demonstrated because to improve health parameters in PLWH.^4^ We previously showed in a pilot study that a 12 week protocol consisting of three sessions per week of 60 min brisk walking at 65–75% of maximal heart rate with (strength‐walk group) 30 min resistance training or without (walk group), improved physical performance, lipid profile, and inflammatory markers.^5^

We assessed body composition and bone health in a subset of 25 cART‐treated PLWH (*Table* S1) from this study [19/25 men, median age: 51 (Q1–Q3: 48–56) years; CD4^+^: 576 (463–701)], with particular attention to the indicators of osteosarcopenia. Percentage of fat mass (FM), fat free mass (FFM) at arms, limbs, and as total body, and bone mineral density (BMD), *t*‐score and *z*‐score at spine (L3/L4), femoral neck, and trochanter were measured by DEXA (Lunar Prodigy, version 8.8, GE, Medical System Madison, WI). The appendicular skeletal muscle mass (ASMMI) index was calculated to assess the presence of sarcopenia (women: ≤5.5 kg/m^2^; men: ≤7.0 kg/m^2^).^6^ The bone *t*‐score was calculated to assess the presence of osteoporosis (−2.5) and osteopenia (−1 to −2.5). Bone remodelling biomarkers were measured in cryopreserved plasma samples by commercially available enzyme‐linked immunosorbent assays (R&D Systems Inc, Minneapolis, MN, USA). These included osteoprotegerin (OPG), receptor activator of NF‐kappaB ligand (RANKL), c‐terminal telopeptide (CTX), and bone alkaline phosphatase (BAP). Samples were analysed in batch at the end of the study and blindly with respect to group assignment. Quantitative variables were expressed as median, first, and third quartiles (Q1–Q3). Per cent changes between baseline (BL) and after 12 week of training W12 within each group were assessed by Wilcoxon signed rank test and between‐groups by Mann–Whitney test.

All participants completed the 12 week programme with a median adherence of 64% (Q1–Q3: 59–75%). Among all participants, significant W12 changes in ASMMI from BL were observed in both training groups, and in total, arms and legs FFM in the strength‐walk group, with changes from BL significantly larger in the strength‐walk than in the walk group for all measurements (*Figure* 1). According to ASMMI, eight participants (32%; three women and five men) had sarcopenia at baseline [women: 5.5 (5.4–5.5) kg/m^2^; men: 6.5 (6.4–6.7) kg/m^2^; age 52 (48–53)]. At Week 12, six of these eight participants normalized ASMMI, including two women [both in the walk group: 6.1 (6.0–6.1) kg/m^2^] and four men [two of the walk group and two of the strength‐walk group: 7.2 (7.1–7.2) kg/m^2^]. Neither significant W12 changes from BL nor significant change differences between groups were observed for BMD, *t*‐score, *z*‐score at spine, femoral neck, and trochanter. According to the *t*‐score, three participants were diagnosed with osteoporosis at BL (12%) and 20 (80%) with osteopenia. At the end of the training protocol, none of the participants improved the *t*‐score (*Table* 1). Significant W12 increases from BL were observed in CTX in both training groups. No significant changes were observed of BAP, OPG and RANKL level, and OPG/RANK ratio (*Table* 1).

**Figure 1:**
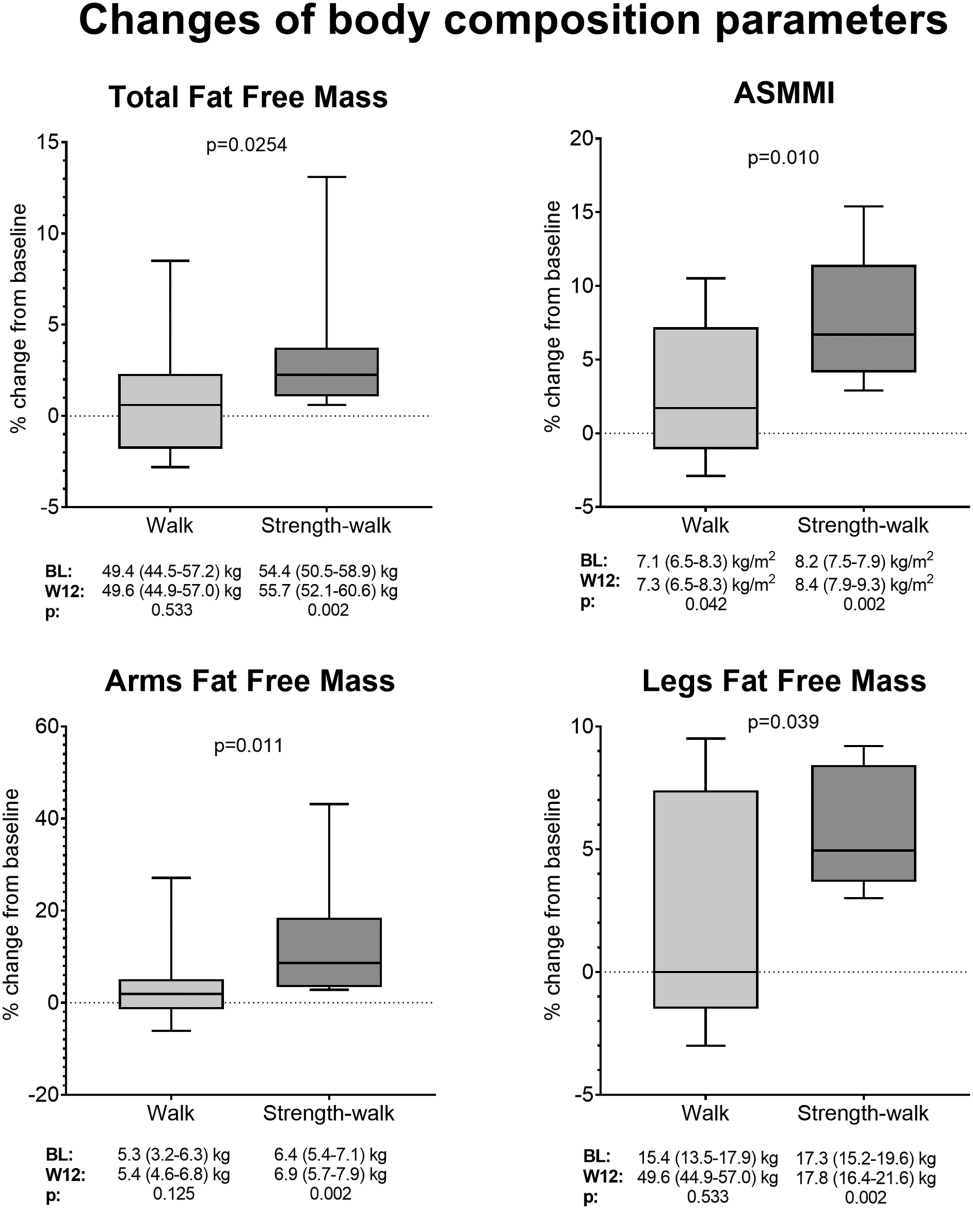
Changes of body composition parameters in people living with HIV following a 12 week physical activity protocol of walk or strength‐walk exercise. Data were assessed by Mann–Whitney test (differences between‐groups) and Wilcoxon signed rank test (changes between BL and W16 within‐groups, below graphs). Level of significance was set at 0.05. Box plots show median and interquartile ranges, and whiskers extend to the highest and lowest observations. ASMMI, appendicular skeletal muscle mass index, BL, baseline; W12, week 12.

**Table 1:** Bone mineral density, *t*‐score, *z*‐score results, and bone remodelling biomarkers results

	Walk (*n* = 15)			Strength‐walk (*n* = 10)		
	BL	W12	*P*	BL	W12	*P*
*Spine*						
BMD (g/cm^2^)	61.5 (56.8–64.8)	62.4 (57.5–65.0)	0.135	62.2 (53.9–64.2)	62.3 (54.5–64.6)	0.109
*t*‐score	−1.2 (−1.6 to –0.8)	−1.1 (−1.5 to –0.7)	0.173	−1.4 (−1.9 to –1.2)	−1.3 (1.8 to –0.9)	0.141
*z*‐score	−1.0 (−1.4 to –0.7)	−0.8 (−1.5 to –0.2)	0.113	−1.4 (−1.8 to –0.8)	−1.2 (−2.0 to –0.7)	0.137
*Femoral neck*						
BMD (g/cm^2^)	4.3 (3.7–5.1)	4.0 (4.8–5.4)	0.107	5.2 (4.3–5.9)	5.2 (4.3–5.9)	0.711
*t*‐score	−1.6 (−1.9 to –0.4)	−1.5 (−1.8 to –0.3)	0.201	−0.9 (−1.0 to –0.5)	−0.8 (−1.1 to –0.4)	0.468
*z*‐score	−1.1 (−1.6 to –0.2)	−0.9 (−1.3 to –0.3)	0.147	−1.3 (−1.5 to –0.8)	−1.1 (−1.5 to –0.2)	0.297
*Trocanther*						
BMD (g/cm^2^)	8.3 (6.7–9.7)	8.6 (6.8–9.8)	0.229	9.7 (8.7–10.3)	9.4 (8.7–10.6)	0.642
*t*‐score	−1.5 (−2–2 to –0.2)	−1.5 (−2.1 to –0.1)	0.108	−0.6 (−1.2 to –0.1)	−0.7 (−1.2 to –0.2)	0.394
*z*‐score	−0.9 (−1.5 to –0.1)	−1.0 (−1.7 to –0.1)	0.688	−0.4 (−0.9 to –0.1)	−0.8 (−1.3 to –0.2)	0.203
*Bone remodelling biomarkers*						
OPG (pg/mL)	1393 (1244–2418)	1617 (1253–2240)	0.107	1324 (957–1658)	1243 (1123–1516)	0.769
RANKL (pg/mL)	4.92 (3.23–7.78)	4.96 (2.62–7.78)	0.497	4.96 (2.62–5.88)	4.36 (2.91–6.57)	0.921
OPG/RANKL	325 (231–492)	354 (195–433)	0.978	228 (173–310)	212 (169–306)	0.193
CTX (pg/mL)	0.54 (0.32–0.84)	0.76 (0.57–0.92)	0.034	0.61 (0.36–0.67)	0.76 (0.46–0.87)	0.005
BAP (ng/mL)	8.44 (5.42–9.71)	7.10 (5.29–9.02)	0.124	8.63 (5.87–11.67)	6.83 (6.12–10.09)	0.185

BAP, bone alkaline phosphatase; BL, baseline; BMD, bone mineral density; CTX, c‐terninal telopeptide; OPG, osteoprotegerin; RANKL, receptor activator of NF‐kappaB ligand; W12, week 12.

Values are expressed as median (Q1–Q3). Data were assessed by Wilcoxon signed rank test (changes between BL and W12 within groups and by BMD).

The main finding of this study was a significant increase in muscle mass in PLWH including patients with sarcopenia. This is relevant because sarcopenia is an emerging health issue in HIV infection,^7^, ^8^ where both ageing and persistent immune activation may contribute to its development, in addition to other potential factors, like cART, risk behaviours, for example, smoking or use of drugs, and other co‐morbidities.^9^ Although moderate aerobic activity alone was also associated in this study with increased appendicular muscle mass, the benefit of concurrent and resistance training was significantly superior, confirming that resistance exercise is relevant to counteract sarcopenia in PLWH.^10^, ^11^ We did not observe improvements of DEXA bone parameters following the 12 week moderate‐intensity exercise protocol. It is possible that this exercise intervention was too short to increase bone mineralization, relative to the length of a bone remodelling cycle.^12^, ^13^ On the other hand, we observed a significant increase of CTX plasma level in both training groups, with neither change of the classical bone formation marker BAP nor of the plasma OPG/RANKL concentration ratio as additional index of bone resorption. The increase of CTX plasma levels following exercise may thus represent a feedback stimulus for the reparative activity of the bone‐remodelling unit.^14^

Our study has some limitations. First, we did not include a non‐exercise control group. Second, the low sample size did not allow drawing firm conclusions on the efficacy of the two training protocols on the studied parameters. Finally, this was a relatively short‐duration study.

In conclusions, PLWH following a physical activity protocol based on the combination of resistance and moderate aerobic training is likely to improve to improve total and appendicular muscle mass. Our study provides information to design larger interventional controlled studies to assess the efficacy of exercise protocols in increasing muscle mass and potentially reducing sarcopenia in PLWH.

## Funding

This work was supported by unrestricted grants from AbbVie and ViiV Healthcare to Associazione Solidarietà AIDS (ASA) and Associazione Nazionale Lotta all'AIDS (ANLAIDS), Italy. This study was supported also by the Italian Ministry of Health (Ricerca Corrente).

## Conflict of interest

The authors have no conflict of interest to declare.

## Supporting information


**Table S1.** Participants' characteristics at baseline.
*Table Note.* Values are either expressed as number of participants (%) or as median (Q1‐Q3). Data were compared between groups by Mann–Whitney and Fisher Exact tests. P values were not significant for all parameters. a: chronic treatment, with no changes during the training period or the 6 weeks before; BMI: Body Mass Index; VACS: Veterans Aging Cohort Study Risk index (this index includes: i) age; ii) laboratory tests: white blood cell count, HIV‐1 RNA, hemoglobin, platelets, AST, ALT, creatinine; iii) liver fibrosis (FIB‐4): composed of AST, ALT, platelets and age; iv) impaired renal function (eGFR): composed of age, gender, race and creatinine; v) HCV status: if the patient ever had a positive antibody test or detectable virus prior the study); NRTI: nucleoside reverse transcriptase inhibitors; NNRTI; non‐nucleoside reverse transcriptase inhibitors; HDL‐C: High density lipoprotein cholesterol; SBP: systolic blood pressure. *Among the inclusion criteria were either objective evidence of lipodystrophy, as established by the visiting physician, or of at least one of the Adult Treatment Panel III definition criteria of the metabolic syndrome.
