# Supplementary material for: Benefits of a 12 week physical activity programme on muscle and bone health in people living with HIV
Source: J Cachexia Sarcopenia Muscle. 2021 Oct 1;12(6):1613–6. doi: 10.1002/jcsm.12824 (PMC8718048; doi:10.1002/jcsm.12824)
Supplement: Supplementary file 1 — Table S1. Participants' characteristics at baseline. Table Note. Values are either expressed as number of participants (%) or as median (Q1‐Q3). Data were compared between groups by Mann–Whitney and Fisher Exact tests. P values were not significant for all parameters. a: chronic treatment, with no changes during the training period or the 6 weeks before; BMI: Body Mass Index; VACS: Veterans Aging Cohort Study Risk index (this index includes: i) age; ii) laboratory tests: white blood cell count, HIV‐1 RNA, hemoglobin, platelets, AST, ALT, creatinine; iii) liver fibrosis (FIB‐4): composed of AST, ALT, platelets and age; iv) impaired renal function (eGFR): composed of age, gender, race and creatinine; v) HCV status: if the patient ever had a positive antibody test or detectable virus prior the study); NRTI: nucleoside reverse transcriptase inhibitors; NNRTI; non‐nucleoside reverse transcriptase inhibitors; HDL‐C: High density lipoprotein cholesterol; SBP: systolic blood pressure. *Among the inclusion criteria were either objective evidence of lipodystrophy, as established by the visiting physician, or of at least one of the Adult Treatment Panel III definition criteria of the metabolic syndrome. [file JCSM-12-1613-s001.docx]

**Table S1.** Participants’ characteristics at baseline.

|  | **All**  **(n=25)** | **Walk**  **(n=15)** | **Strenght-walk**  **(n=10)** |
| --- | --- | --- | --- |
| **Demographic and general characteristics**  Male gender (n, %)  Age (years, median, Q1-Q3)  BMI (kg/m^2^, median, Q1-Q3)  Smokers (n, %) | 20 (80%)  51 (48-56)  26 (23-28)  7 (28%) | 10 (60%)  51 (48-55)  26 (22-29)  5 (33%) | 10 (100%)  53 (47-59)  25 (22-27)  2 (20%) |
| **HIV Infection Risk Group**  Ex-intravenous drug users (n, %)  Men-having-Sex-with-Men (n, %)  Heterosexual (n, %)  Vertical (n, %) | 7 (28%)  13 (52%)  4 (16%)  1 (4%) | 6 (40%)  4 (27%)  4 (27%)  1 (6%) | 1 (10%)  9 (90%)  0  0 |
| **HIV Infection Variables**  Nadir CD4^+^ (T-cells/μL)  Current CD4^+^ (T-cells/μL)  Viral Load (<40 c/mL)  VACS Index (median, Q1-Q3) | 145 (38-197)  577 (463-701)  24 (96%)  23 (9-35) | 75 (31-154)  594 (430-716)  15 (100%)  22 (16-31) | 71 (32-93)  704 (516-881)  9 (90%)  10 (6-12) |
| **HIV Infection and other Treatments**  2NRTI + Protease Inhibitor (n, %)  2NRTI + NNRTI (n, %)  Other cART regimens (n, %)  Beta blockers (n, %) ^a^  Other anti-hypertensive drugs (n, %) ^a^  Statins (n, %) ^a^  Fibrates (n, %) ^a^ | 10 (40%)  8 (32%)  7 (28%)  4 (16%)  8 (32%)  3 (12%)  2 (8%) | 6 (40%)  5 (33%)  4 (27%)  3 (20%)  5 (33%)  3 (20%)  2 (13%) | 4 (40%)  3 (30%)  3 (30%)  1 (10%)  3 (30%)  0  0 |
| **Inclusion criteria***  Lipodistrophy  ≥ 1 metabolic syndrome criterion  Blood triglycerides ≥ 150 mg/dL  Blood HDL-C ≤ 40 (M) or ≤ 50 (W) mg/dL  Blood glucose ≥ 110 mg/dL  Waist ≥ 102 (M) or 88 (W)  SBP ≥ 150 or DBP ≥ 85 mmHg | 25 (100%)  15 (60%)  8 (32%)  9 (36%)  1 (4%)  7 (28%)  5 (20%) | 15 (100%)  9 (60%)  4 (27%)  6 (40%)  0  6 (40%)  4 (27%) | 10 (100%)  6 (60%)  4 (40%)  3 (30%)  1 (10%)  1 (10%)  1 (10%) |

*Table Note.* Values are either expressed as number of participants (%) or as median (Q1-Q3). Data were compared between groups by Mann-Whitney and Fisher Exact tests. P values were not significant for all parameters. a: chronic treatment, with no changes during the training period or the 6 weeks before; BMI: Body Mass Index; VACS: Veterans Aging Cohort Study Risk index (this index includes: i) age; ii) laboratory tests: white blood cell count, HIV-1 RNA, hemoglobin, platelets, AST, ALT, creatinine; iii) liver fibrosis (FIB-4): composed of AST, ALT, platelets and age; iv) impaired renal function (eGFR): composed of age, gender, race and creatinine; v) HCV status: if the patient ever had a positive antibody test or detectable virus prior the study); NRTI: nucleoside reverse transcriptase inhibitors; NNRTI; non-nucleoside reverse transcriptase inhibitors; HDL-C: High density lipoprotein cholesterol; SBP: systolic blood pressure. *Among the inclusion criteria were either objective evidence of lipodystrophy, as established by the visiting physician, or of at least one of the Adult Treatment Panel III definition criteria of the metabolic syndrome.
